# Supplementary figures and images for: Systemic GFP silencing is associated with high transgene expression in Nicotiana benthamiana
Source: PLoS One. 2021 Mar 15;16(3):e0245422. doi: 10.1371/journal.pone.0245422 (PMC7959375; doi:10.1371/journal.pone.0245422)

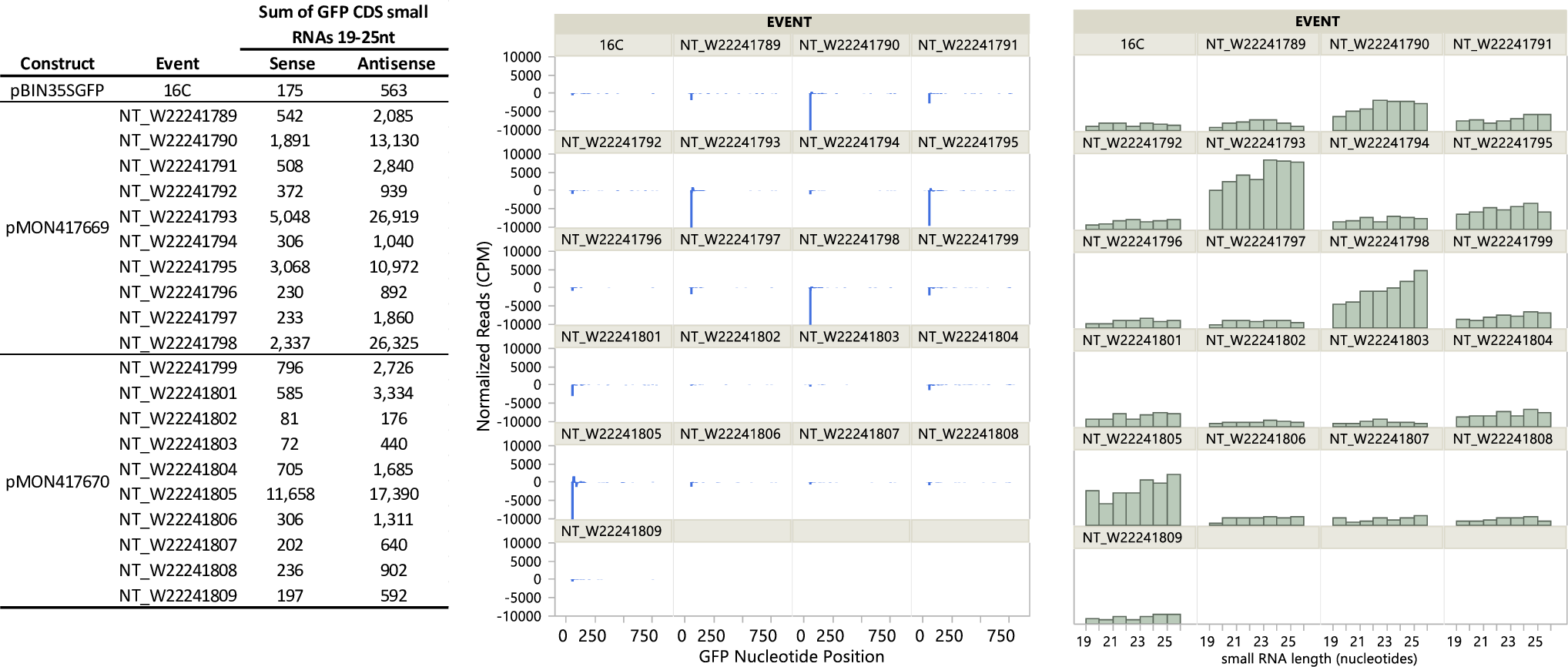

Supplement: S1 Fig — Untreated tissue from the first true leaf of each homozygous R2 transgenic events was sampled and the small RNAs were sequenced. The experiment was arranged as a randomized complete block with 4 replications per treatment. The replicates for each treatment were pooled prior to small RNA sequencing. The sequencing data are expressed as the sum of small RNA counts 19-25nt in length per 1x10^6^ total small RNA reads. (TIF) [file pone.0245422.s001.tif]
