# Supplementary material for: Systemic GFP silencing is associated with high transgene expression in Nicotiana benthamiana
Source: PLoS One. 2021 Mar 15;16(3):e0245422. doi: 10.1371/journal.pone.0245422 (PMC7959375; doi:10.1371/journal.pone.0245422)
Supplement: S1 Table — (DOCX) [file pone.0245422.s002.docx]

| **Name** | **Sequence** |
| --- | --- |
| Nb.CHeH_F | GCT AAT GCT CAG GTA CGA ACG |
| Nb.CHeH_R | CTC AAT CTC ACG AAC TCC CTC |
| Nb.CheH_Probe | /56-FAM/TGC ATC AAG /ZEN/CCT CAC AGT CTC GG/3IABkFQ/ |
| 16C_GFP_F | CGA CGG GAA CTA CAA GAC AC |
| 16C_GFP_R | TTA AGC TCG ATC CTG TTG ACG |
| 16C_GFP_Probe | /56-FAM/TC TCC CTC A/Zen/A ACT TGA CTT CAG CAC G/3IABkFQ/ |
| Nb.PP2a_F | AAC TAT GTG AAG CTG TCG GG |
| Nb.PP2a_R | TCT CGA AGC AAA CGG ACA TAG |
| Nb.PP2a_Probe | /5TET/AC TAG GAC G/Zen/G ATT TGG TGC CTG C/3IABkFQ/ |
