# Supplementary material for: Systemic GFP silencing is associated with high transgene expression in Nicotiana benthamiana
Source: PLoS One. 2021 Mar 15;16(3):e0245422. doi: 10.1371/journal.pone.0245422 (PMC7959375; doi:10.1371/journal.pone.0245422)
Supplement: S2 Table — (DOCX) [file pone.0245422.s003.docx]

|  | **Sequence (5'-3')** | |
| --- | --- | --- |
| **Gene Target** | **Sense** | **Antisense** |
| 16C_GFP | GGCAUCAAAGCCAACUUCAAAA | UUGAAGUUGGCUUUGAUGCCGU |
| Nb_MgChl-H | AUCAUGGAAUUGGAGGCAAAAG | UUUGCCUCCAAUUCCAUGAUCA |
