# Supplementary material for: Systemic GFP silencing is associated with high transgene expression in Nicotiana benthamiana
Source: PLoS One. 2021 Mar 15;16(3):e0245422. doi: 10.1371/journal.pone.0245422 (PMC7959375; doi:10.1371/journal.pone.0245422)
Supplement: S1 File — (PDF) [file pone.0245422.s004.pdf]

>pMON417669_InsertOnly(NPT+GFP+tnpA)
GCGGCCGCCAATCTGATCATGAGCGGAGAATTAAGGGAGTCACGTTATGACCCCCGCCGATGACGCGGGACAAGCCGTTTT
ACGTTTGGAACTGACAGAACCGCAACGTTGAAGGAGCCACTCAGCCGCGGGTTTCTGGAGTTTAATGAGCTAAGCACATACG
TCAGAAACCATTATTGCGCGTTCAAAAGTCGCCTAAGGTCACTATCAGCTAGCAAATATTTCTTGTCAAAAATGCTCCACTGAC
GTTCCATAAATTCCCCTCGGTATCCAATTAGAGTCTCATATTCACTCTCAATCCAAATAATCTGCACCGGATCTGGATCGTTTCG
CATGATTGAACAAGATGGATTGCACGCAGGTTCTCCGGCCGCTTGGGTGGAGAGGCTATTCGGCTATGACTGGGCACAACA
GACAATCGGCTGCTCTGATGCCGCCGTGTTCCGGCTGTCAGCGCAGGGGCGCCCGGTTCTTTTTGTCAAGACCGACCTGTCC
GGTGCCCTGAATGAACTGCAGGACGAGGCAGCGCGGCTATCGTGGCTGGCCACGACGGGCGTTCCTTGCGCAGCTGTGCTC
GACGTTGTCACTGAAGCGGGAAGGGACTGGCTGCTATTGGGCGAAGTGCCGGGGCAGGATCTCCTGTCATCTCACCTTGCTC
CTGCCGAGAAAGTATCCATCATGGCTGATGCAATGCGGCGGCTGCATACGCTTGATCCGGCTACCTGCCCATTCGACCACCA
AGCGAAACATCGCATCGAGCGAGCACGTACTCGGATGGAAGCCGGTCTTGTCGATCAGGATGATCTGGACGAAGAGCATCA
GGGGCTCGCGCCAGCCGAACTGTTCGCCAGGCTCAAGGCGCGCATGCCCGACGGCGATGATCTCGTCGTGACCCATGGCGA
TGCCTGCTTGCCGAATATCATGGTGGAAAATGGCCGCTTTTCTGGATTCATCGACTGTGGCCGGCTGGGTGTGGCGGACCGC
TATCAGGACATAGCGTTGGCTACCCGTGATATTGCTGAAGAGCTTGGCGGCGAATGGGCTGACCGCTTCCTCGTGCTTTACG
GTATCGCCGCTCCCGATTCGCAGCGCATCGCCTTCTATCGCCTTCTTGACGAGTTCTTCTGAGCGGGACTCTGGGGTTCGAAA
TGACCGACCAAGCGACGCCCAACCTGCCATCACGAGATTTCGATTCCACCGCCGCCTTCTATGAAAGGTTGGGCTTCGGAATC
GTTTTCCGGGACGCCGGCTGGATGATCCTCCAGCGCGGGGATCTCATGCTGGAGTTCTTCGCCCACGGGATCTCTGCGGAAC
AGGCGGTCGAAGGTGCCGATATCATTACGACAGCAACGGCCGACAAGCACAACGCCACGATCCTGAGCGACAATATGATCG
GGCCCGGCGTCCACATCAACGGCGTCGGCGGCGACTGCCCAGGCAAGACCGAGATGCACCGCGATATCTTGCTGCGTTCGG
ATATTTTCGTGGAGTTCCCGCCACAGACCCGGATGATCCCCGATCGTTCAAACATTTGGCAATAAAGTTTCTTAAGATTGAATC
CTGTTGCCGGTCTTGCGATGATTATCATATAATTTCTGTTGAATTACGTTAAGCATGTAATAATTAACATGTAATGCATGACGT
TATTTATGAGATGGGTTTTTATGATTAGAGTCCCGCAATTATACATTTAATACGCGATAGAAAACAAAATATAGCGCGCAAAC
TAGGATAAATTATCGCGCGCGGTGTCATCTATGTTACTAGATCGGGCCTCCTGTCAATGCTGGCGGCGGCTCTGGTGGTGGT
TCTGGTGGCGGCTCTGAGGGTGGTGGCTCTGAGGGTGGCGGTTCTGAGGGTGGCGGCTCTGAGGGAGGCGGTTCCGGTGG
TGGCTCTGGTTCCGGTGATTTTGATTATGAAAAGATGGCAAACGCTAATAAGGGGGCTATGACCGAAAATGCCGATGAAAAC
GCGCTACAGTCTGACGCTAAAGGCAAACTTGATTCTGTCGCTACTGATTACGGTGCTGCTATCGATGGTTTCATTGGTGACGT
TTCCGGCCTTGCTAATGGTAATGGTGCTACTGGTGATTTTGCTGGCTCTAATTCCCAAATGGCTCAAGTCGGTGACGGTGATA
ATTCACCTTTAATGAATAATTTCCGTCAATATTTACCTTCCCTCCCTCAATCGGTTGAATGTCGCCCTTTTGTCTTTGGCCCAATA
CGCAAACCGCCTCTCCCCGCGCGTTGGCCGATTCATTAATGCAGCTGGCACGACAGGTTTCCCGACTGGAAAGCGGGCAGTG
AGCGCAACGCAATTAATGTGAGTTAGCTCACTCATTAGGCACCCCAGGCTTTACACTTTATGCTTCCGGCTCGTATGTTGTGT
GGAATTGTGAGCGGATAACAATTTCACACAGGAAACAGCTATGACCATGATTACGCCAAGCTTGCATGCCTGCAGGTCCCCA
GATTAGCCTTTTCAATTTCAGAAAGAATGCTAACCCACAGATGGTTAGAGAGGCTTACGCAGCAGGTCTCATCAAGACGATCT
ACCCGAGCAATAATCTCCAGGAAATCAAATACCTTCCCAAGAAGGTTAAAGATGCAGTCAAAAGATTCAGGACTAACTGCAT
CAAGAACACAGAGAAAGATATATTTCTCAAGATCAGAAGTACTATTCCAGTATGGACGATTCAAGGCTTGCTTCACAAACCAA
GGCAAGTAATAGAGATTGGAGTCTCTAAAAAGGTAGTTCCCACTGAATCAAAGGCCATGGAGTCAAAGATTCAAATAGAGG
ACCTAACAGAACTCGCCGTAAAGACTGGCGAACAGTTCATACAGAGTCTCTTACGACTCAATGACAAGAAGAAAATCTTCGT
CAACATGGTGGAGCACGACACACTTGTCTACTCCAAAAATATCAAAGATACAGTCTCAGAAGACCAAAGGGCAATTGAGACT
TTTCAACAAAGGGTAATATCCGGAAACCTCCTCGGATTCCATTGCCCAGCTATCTGTCACTTTATTGTGAAGATAGTGGAAAA
GGAAGGTGGCTCCTACAAATGCCATCATTGCGATAAAGGAAAGGCCATCGTTGAAGATGCCTCTGCCGACAGTGGTCCCAAA
GATGGACCCCCACCCACGAGGAGCATCGTGGAAAAAGAAGACGTTCCAACCACGTCTTCAAAGCAAGTGGATTGATGTGAT
ATCTCCACTGACGTAAGGGATGACGCACAATCCCACTATCCTTCGCAAGACCCTTCCTCTATATAAGGAAGTTCATTTCATTTG
GAGAGAACACGGGGGACTCTAGAGGATCCAAGGAGATATAACAATGAAGACTAATCTTTTTCTCTTTCTCATCTTTTCACTTC
TCCTATCATTATCCTCGGCCGAATTCAGTAAAGGAGAAGAACTTTTCACTGGAGTTGTCCCAATTCTTGTTGAATTAGATGGT
GATGTTAATGGGTACAAATTTTCTGTCAGTGGAGAGGGTGAAGGTGATGCAACATACGGAAAACTTACCCTTAAATTTATTT
GCACTACTGGAAAACTACCTGTTCCATGGCCAACACTTGTCACTACTTTCTCTTATGGTGTTCAATGCTTTTCAAGATACCCAG
ATCATATGAAGCGGCACGACTTCTTCAAGAGCGCCATGCCTGAGGGATACGTGCAGGAGAGGACCATCTTCTTCAAGGACG
ACGGGAACTACAAGACACGTGCTGAAGTCAAGTTTGAGGGAGACACCCTCGTCAACAGGATCGAGCTTAAGGGAATCGATT
TCAAGGAGGACGGAAACATCCTCGGCCACAAGTTGGAATACAACTACAACTCCCACAACGTATACATCATGGCCGACAAGCA
AAAGAACGGCATCAAAGCCAACTTCAAGACCCGCCACAACATCGAAGACGGCGGCGTGCAACTCGCTGATCATTATCAACAA
AATACTCCAATTGGCGATGGCCCTGTCCTTTTACCAGACAACCATTACCTGTCCACACAATCTGCCCTTTCGAAAGATCCCAAC
GAAAAGAGAGACCACATGGTCCTTCTTGAGTTTGTAACAGCTGCTGGGATTACACATGGCATGGATGAACTATACAAACATG
ATGAGCTTTAAGAGCTCGAATTTCCCCGATCGTTCAAACATTTGGCAATAAAGTTTCTTAAGATTGAATCCTGTTGCCGGTCTT

GCGATGATTATCATATAATTTCTGTTGAATTACGTTAAGCATGTAATAATTAACATGTAATGCATGACGTTATTTATGAGATGG
GTTTTTATGATTAGAGTCCCGCAATTATACATTTAATACGCGATAGAAAACAAAATATAGCGCGCAAACTAGGATAAATTATC
GCGCGCGGTGTCATCTATGTTACTAGATCGGGAATTAATTCACTGGCCGTCGTTTTACAACGTCGTGACTGGGAAAACCCTG
GCGTTACCCAACTTAATCGCCTTGCAGCACATCCCCCTTTCGCCAGCTGGCGTAATAGCGAAGAGGCCCGCACCGATCGCCCT
TCCCAACAGTTGCGCAGCCTGAATGGCGCCCGCTCCTTTCGCTTTCTTCCCTTCCTTTCTCGCCACGTTGGGGTCGTTTGCGGG
AGGGGGCGGAATCCTACGCTAAGGCTTTGGCCAGCGATATTCTCCGGTGAGATTGATGTGTTCCCATCCGAGCGGCGAAACA
TGGGCCAAGAGATCGGGCGATAGCAGCTTTCCATCGCGTTTCTGGTTTGCAACGACCTCGCCGAGCTTCATGGTGTTCCAGA
AGATGATGATGGCGGCGAGCAGATTCATGCCGGCGATGCGGTAATGCTGGCCTTCGGCGGAACGGTCGCGGATTTCACCGC
GGCGGTGGAAGCTGATTGCCCGCTTCAGCGCATGATGAGCTTCGCCTTTGTTGAGCCCGATCTGGGCACGCCGTTGGAGTTC
GGCATCCAGAATCCAGTCGATCATGAACAGGGTGCGCTCGACGCGACCGACTTCCCGCAGGGCTGTCGCGAGCTCGTTCTGC
CGCGGATAGGAGGCGAGTTTCCGCAGAATCTGGCTTGGCGCGACGGTCCCGGCAGCAATGGTGGCGGCGATGCGCAGGAT
GTCGGGCCAATTGCGCTCGATCATGGCTTGGTTGACCTTTCCGCCGATCAACGCTCGCAGGTGCGCCGGGGCGGCCGACGG
ATTGAACGCGTAGAGCCGTTTGGATGGCAGGTCGCGGATGCGCGGAGCGAACCGGTAGCCGAGAATGGCACATGCGGCAA
AGACGTGATCGGTGAAGCCGCCCGTGTCGGTGAACTGCTCGCGGATATGGCGTCCAGCATCGTTCATCAGCAGGCCATCGA
GGATGTAAGGCGCTTCGCTTGCCGTTGCAGGAATCACCTGGGTTGCGAACGGCGCATATTGGTCGGAGACGTGGCTATAGG
CTTTCAGGCCCGGGGTATTGCCATATTTCGCGTTGACCAGGTTCATGGCCTCACCTTGCTCTGTAGCGACGAAGAACTGTCCG
TCGCTCGAAGCCGACGTGCCCATGCCCCAGAACCGGGCCATGGGTAACGCTGCCTGTGCCTCGACCACCATGGCCAGCGCCC
GGTCATAGGCTTCGCCCTCGACATGCCACCGTCCAATGCGGATCAATTCCCAGAAGGTGTGGGTGTTTGTCGCATCCGCCATT
TTGCGCAAGCCGAGGTTGATCCCTTCCGCCAAGATAACGTTCATTAGCCCGATCCGGTCAGCGCAGGGTGCTCCTGTGCGCA
GATGGGTGAACGCTTCGGTGAAGCCGGTCGCCGCATCCACCTCCAGCAGGAGATCGGTGATGCGCGTGGGCGGGATCTGCT
TGTAGAGATCGAGCACCAGATCTTCGGCGCCTGTCGGCGCGGCGGCTTCGAGTTTCTCGATATGCAGAACGCCGTTTTCAAT
CGACCCGCCCGGGATCGTGCCTGCGCGAGCGGCACGGCCAAGCTCGCGCAACCGCATGTCGAGGCGAGCTTGCCGGTCTGC
CAGCCATTCCTCCGGCCGCAATGGCACAGCGAGACGACCGCCTTCCGCGATGGATTGTGCCGGAACGAGTGCGTGTTTCAGA
TCGCCATAGCGCCGGGACCTAGTAAGCCAGACATCTCCGGAGCGGAACGCATCGCGCAGATGGAACAGCACCGCGATCTCC
CATAGGCGAGCGTCGCCAGCCCTCTGGGCCCGAAGGTGGCGATGCCATTTCGAGCTGGGCCGCAAGAAGCTGGTCATCGCG
GCATCGTTCAAACCGGTACGAAGGGCCGTCACCGCTTCCAGAAGCGGCAGTGCAACGGGCGCAGCTCGCAGATCGAGCAGG
CGCAACATGCGTGGAGCGTATCGGCGGAAGCGGTGATAACCGTCGAGCACATGATTGAGCGGATCGTCGGCCATGGTGGC
GGTCAGCCTGGTTGCCATTGCAACAAGGGTTTTTAAGCCGTCCCACCCTGACCCACTCGCGATGACATCGCCCAGCGGCTGG
CCATCATCCTGTGCATCGACCAGGGCGCCCCCGATCTCGGCGAAGGATTTCAGGGTGTCACGCACCACCCCCGCTTCGTCTGC
GACCTTTGCATGGCAAATACGCTCCGAAGCACGGTAGAGACGGCCGACGATCCGGTCGTGGGTTTCGACCACTGCGTCGGC
CAACATCGCCTGCCATTCCGAGACGCAAACAGCCAAGATCGCAAGCCGCCTGTCCTCCGGGAGATCGCGCATGCCGTCGGCA
TAATACCGTTCACCCTGCCTGCGCAGACGAGTCACCCGATGGGCAGGAACGCCGGCAAGCAGATCCTCGGGGAGATCGATG
CGTTGCAGATATTCGAGCCGGTCGAGCAGCCGGTTGGCCGACGAAGAGTTCGAGCCAGGCTCGAACTGGCGCAGCCACACA
AAACGGGTCACCCGATCATCAGCCGTCTCCTCGAGCAATGCCAGCAACTGTTCTCGGATCGACATAGGCAGCCGACTGGCGA
TCCTCGTCTCGATGCGTCGCTCGGCATCGACGAGAGCCGCGGCACAAAGCCGCTCGATCGTGGATGTCGCGGGAAGGACAG
TGCGGGTGCGTCGGCACTCGGCTACGAAGCGACGGGCGATATCCTCGTTCGACACCGCCATCTCGGCTTCTCGGAACAACCA
TTCCTTCAGCTCGCTCGCACCACGTCCGGAGAAGGTGCGGAAGCCGTAGAGCCCCCGTAACTCGGCAAGATGCTCGTGCCGT
GTTTCCTCGCGGGCAGCATAGTCTACGAGATCGTCGGCACCCAGGCCAAGCTGCGCTCCGATAAATTCGATGACCTCTGCAG
GGATCAGTTCGCCTGGAGCCAGCACCCGGCCGGGATAGCGCAGGACACACAATTGCAGGGCGAAGCCGAACCTGTTGTGA
GCGCGCCGACGCAGCCTGATATGCCCAAGGTCTTCATCACTCAGCGTATAGTGCTTGAGCAAATCCGTCTGTGAAGTCGGCA
AGCGCAACAGCGCGTCTTTCTGCCGATCGGTTAGAGTGACGCGACGCGGCATACATGTTCCTTTTTCAAAATCTGATAGCGTT
CAAGACGCTTTGTTTATGAAGCTGGTTGAGATACATTTCCAGAGGTCAATGCAATCGTGGCCGAAGCGCCGCCTCAAACCAA
CGTTTGTGATACATGCTGATCGGATATGCCCGCGTCTCCAAAGCCGATGGCTCGCAGTCTCTCGACCTGCAGCACGACGCCTT
GCGCGCCGCAGGTGTCGAACGGGACAATATCTATGATGATCTTGCTTCCGGCGGTCGTGATGATCGCCCTGGCTTGACTGCC
TGCCTCAAGTCATTGCGTGACGGCGATGTGCTGGTGGTCTGAAAACTAGT

>pMON417670_InsertOnly(NPTII+GFP)
GCGGCCGCCAATCTGATCATGAGCGGAGAATTAAGGGAGTCACGTTATGACCCCCGCCGATGACGCGGGACAAGCCGTTTT
ACGTTTGGAACTGACAGAACCGCAACGTTGAAGGAGCCACTCAGCCGCGGGTTTCTGGAGTTTAATGAGCTAAGCACATACG
TCAGAAACCATTATTGCGCGTTCAAAAGTCGCCTAAGGTCACTATCAGCTAGCAAATATTTCTTGTCAAAAATGCTCCACTGAC
GTTCCATAAATTCCCCTCGGTATCCAATTAGAGTCTCATATTCACTCTCAATCCAAATAATCTGCACCGGATCTGGATCGTTTCG
CATGATTGAACAAGATGGATTGCACGCAGGTTCTCCGGCCGCTTGGGTGGAGAGGCTATTCGGCTATGACTGGGCACAACA
GACAATCGGCTGCTCTGATGCCGCCGTGTTCCGGCTGTCAGCGCAGGGGCGCCCGGTTCTTTTTGTCAAGACCGACCTGTCC

GGTGCCCTGAATGAACTGCAGGACGAGGCAGCGCGGCTATCGTGGCTGGCCACGACGGGCGTTCCTTGCGCAGCTGTGCTC
GACGTTGTCACTGAAGCGGGAAGGGACTGGCTGCTATTGGGCGAAGTGCCGGGGCAGGATCTCCTGTCATCTCACCTTGCTC
CTGCCGAGAAAGTATCCATCATGGCTGATGCAATGCGGCGGCTGCATACGCTTGATCCGGCTACCTGCCCATTCGACCACCA
AGCGAAACATCGCATCGAGCGAGCACGTACTCGGATGGAAGCCGGTCTTGTCGATCAGGATGATCTGGACGAAGAGCATCA
GGGGCTCGCGCCAGCCGAACTGTTCGCCAGGCTCAAGGCGCGCATGCCCGACGGCGATGATCTCGTCGTGACCCATGGCGA
TGCCTGCTTGCCGAATATCATGGTGGAAAATGGCCGCTTTTCTGGATTCATCGACTGTGGCCGGCTGGGTGTGGCGGACCGC
TATCAGGACATAGCGTTGGCTACCCGTGATATTGCTGAAGAGCTTGGCGGCGAATGGGCTGACCGCTTCCTCGTGCTTTACG
GTATCGCCGCTCCCGATTCGCAGCGCATCGCCTTCTATCGCCTTCTTGACGAGTTCTTCTGAGCGGGACTCTGGGGTTCGAAA
TGACCGACCAAGCGACGCCCAACCTGCCATCACGAGATTTCGATTCCACCGCCGCCTTCTATGAAAGGTTGGGCTTCGGAATC
GTTTTCCGGGACGCCGGCTGGATGATCCTCCAGCGCGGGGATCTCATGCTGGAGTTCTTCGCCCACGGGATCTCTGCGGAAC
AGGCGGTCGAAGGTGCCGATATCATTACGACAGCAACGGCCGACAAGCACAACGCCACGATCCTGAGCGACAATATGATCG
GGCCCGGCGTCCACATCAACGGCGTCGGCGGCGACTGCCCAGGCAAGACCGAGATGCACCGCGATATCTTGCTGCGTTCGG
ATATTTTCGTGGAGTTCCCGCCACAGACCCGGATGATCCCCGATCGTTCAAACATTTGGCAATAAAGTTTCTTAAGATTGAATC
CTGTTGCCGGTCTTGCGATGATTATCATATAATTTCTGTTGAATTACGTTAAGCATGTAATAATTAACATGTAATGCATGACGT
TATTTATGAGATGGGTTTTTATGATTAGAGTCCCGCAATTATACATTTAATACGCGATAGAAAACAAAATATAGCGCGCAAAC
TAGGATAAATTATCGCGCGCGGTGTCATCTATGTTACTAGATCGGGCCTCCTGTCAATGCTGGCGGCGGCTCTGGTGGTGGT
TCTGGTGGCGGCTCTGAGGGTGGTGGCTCTGAGGGTGGCGGTTCTGAGGGTGGCGGCTCTGAGGGAGGCGGTTCCGGTGG
TGGCTCTGGTTCCGGTGATTTTGATTATGAAAAGATGGCAAACGCTAATAAGGGGGCTATGACCGAAAATGCCGATGAAAAC
GCGCTACAGTCTGACGCTAAAGGCAAACTTGATTCTGTCGCTACTGATTACGGTGCTGCTATCGATGGTTTCATTGGTGACGT
TTCCGGCCTTGCTAATGGTAATGGTGCTACTGGTGATTTTGCTGGCTCTAATTCCCAAATGGCTCAAGTCGGTGACGGTGATA
ATTCACCTTTAATGAATAATTTCCGTCAATATTTACCTTCCCTCCCTCAATCGGTTGAATGTCGCCCTTTTGTCTTTGGCCCAATA
CGCAAACCGCCTCTCCCCGCGCGTTGGCCGATTCATTAATGCAGCTGGCACGACAGGTTTCCCGACTGGAAAGCGGGCAGTG
AGCGCAACGCAATTAATGTGAGTTAGCTCACTCATTAGGCACCCCAGGCTTTACACTTTATGCTTCCGGCTCGTATGTTGTGT
GGAATTGTGAGCGGATAACAATTTCACACAGGAAACAGCTATGACCATGATTACGCCAAGCTTGCATGCCTGCAGGTCCCCA
GATTAGCCTTTTCAATTTCAGAAAGAATGCTAACCCACAGATGGTTAGAGAGGCTTACGCAGCAGGTCTCATCAAGACGATCT
ACCCGAGCAATAATCTCCAGGAAATCAAATACCTTCCCAAGAAGGTTAAAGATGCAGTCAAAAGATTCAGGACTAACTGCAT
CAAGAACACAGAGAAAGATATATTTCTCAAGATCAGAAGTACTATTCCAGTATGGACGATTCAAGGCTTGCTTCACAAACCAA
GGCAAGTAATAGAGATTGGAGTCTCTAAAAAGGTAGTTCCCACTGAATCAAAGGCCATGGAGTCAAAGATTCAAATAGAGG
ACCTAACAGAACTCGCCGTAAAGACTGGCGAACAGTTCATACAGAGTCTCTTACGACTCAATGACAAGAAGAAAATCTTCGT
CAACATGGTGGAGCACGACACACTTGTCTACTCCAAAAATATCAAAGATACAGTCTCAGAAGACCAAAGGGCAATTGAGACT
TTTCAACAAAGGGTAATATCCGGAAACCTCCTCGGATTCCATTGCCCAGCTATCTGTCACTTTATTGTGAAGATAGTGGAAAA
GGAAGGTGGCTCCTACAAATGCCATCATTGCGATAAAGGAAAGGCCATCGTTGAAGATGCCTCTGCCGACAGTGGTCCCAAA
GATGGACCCCCACCCACGAGGAGCATCGTGGAAAAAGAAGACGTTCCAACCACGTCTTCAAAGCAAGTGGATTGATGTGAT
ATCTCCACTGACGTAAGGGATGACGCACAATCCCACTATCCTTCGCAAGACCCTTCCTCTATATAAGGAAGTTCATTTCATTTG
GAGAGAACACGGGGGACTCTAGAGGATCCAAGGAGATATAACAATGAAGACTAATCTTTTTCTCTTTCTCATCTTTTCACTTC
TCCTATCATTATCCTCGGCCGAATTCAGTAAAGGAGAAGAACTTTTCACTGGAGTTGTCCCAATTCTTGTTGAATTAGATGGT
GATGTTAATGGGTACAAATTTTCTGTCAGTGGAGAGGGTGAAGGTGATGCAACATACGGAAAACTTACCCTTAAATTTATTT
GCACTACTGGAAAACTACCTGTTCCATGGCCAACACTTGTCACTACTTTCTCTTATGGTGTTCAATGCTTTTCAAGATACCCAG
ATCATATGAAGCGGCACGACTTCTTCAAGAGCGCCATGCCTGAGGGATACGTGCAGGAGAGGACCATCTTCTTCAAGGACG
ACGGGAACTACAAGACACGTGCTGAAGTCAAGTTTGAGGGAGACACCCTCGTCAACAGGATCGAGCTTAAGGGAATCGATT
TCAAGGAGGACGGAAACATCCTCGGCCACAAGTTGGAATACAACTACAACTCCCACAACGTATACATCATGGCCGACAAGCA
AAAGAACGGCATCAAAGCCAACTTCAAGACCCGCCACAACATCGAAGACGGCGGCGTGCAACTCGCTGATCATTATCAACAA
AATACTCCAATTGGCGATGGCCCTGTCCTTTTACCAGACAACCATTACCTGTCCACACAATCTGCCCTTTCGAAAGATCCCAAC
GAAAAGAGAGACCACATGGTCCTTCTTGAGTTTGTAACAGCTGCTGGGATTACACATGGCATGGATGAACTATACAAACATG
ATGAGCTTTAAGAGCTCGAATTTCCCCGATCGTTCAAACATTTGGCAATAAAGTTTCTTAAGATTGAATCCTGTTGCCGGTCTT
GCGATGATTATCATATAATTTCTGTTGAATTACGTTAAGCATGTAATAATTAACATGTAATGCATGACGTTATTTATGAGATGG
GTTTTTATGATTAGAGTCCCGCAATTATACATTTAATACGCGATAGAAAACAAAATATAGCGCGCAAACTAGGATAAATTATC
GCGCGCGGTGTCATCTATGTTACTAGATCGGGAATTAATTCACTGGCCGTCGTTTTACAACGTCGTGACTGGGAAAACCCTG
GCGTTACCCAACTTAATCGCCTTGCAGCACATCCCCCTTTCGCCAGCTGGCGTAATAGCGAAGAGGCCCGCACCGATCGCCCT
TCCCAACAGTTGCGCAGCCTGAATGGCGCCCGCTCCTTTCGCTTTCTTCCCTTCCTTTCTCGCCACGTTACTAGT
